# The Role of Interoceptive Sensibility and Emotional Conceptualization for the Experience of Emotions

**DOI:** 10.3389/fpsyg.2021.712418

**Published:** 2021-11-18

**Authors:** Carlos Ventura-Bort, Julia Wendt, Mathias Weymar

**Affiliations:** ^1^Department of Biological Psychology and Affective Science, Faculty of Human Sciences, University of Potsdam, Potsdam, Germany; ^2^Faculty of Health Sciences, Brandenburg Medical School, University of Potsdam, Potsdam, Germany

**Keywords:** emotion, granularity, emotional intensity, well-being, adaptability, interoceptive sensibility, interoception

## Abstract

The theory of constructed emotions suggests that different psychological components, including core affect (mental and neural representations of bodily changes), and conceptualization (meaning-making based on prior experiences and semantic knowledge), are involved in the formation of emotions. However, little is known about their role in experiencing emotions. In the current study, we investigated how individual differences in interoceptive sensibility and emotional conceptualization (as potential correlates of these components) interact to moderate three important aspects of emotional experiences: emotional intensity (strength of emotion felt), arousal (degree of activation), and granularity (ability to differentiate emotions with precision). To this end, participants completed a series of questionnaires assessing interoceptive sensibility and emotional conceptualization and underwent two emotion experience tasks, which included standardized material (emotion differentiation task; ED task) and self-experienced episodes (day reconstruction method; DRM). Correlational analysis showed that individual differences in interoceptive sensibility and emotional conceptualization were related to each other. Principal Component Analysis (PCA) revealed two independent factors that were referred to as sensibility and monitoring. The Sensibility factor, interpreted as beliefs about the accuracy of an individual in detecting internal physiological and emotional states, predicted higher granularity for negative words. The Monitoring factor, interpreted as the tendency to focus on the internal states of an individual, was negatively related to emotional granularity and intensity. Additionally, Sensibility scores were more strongly associated with greater well-being and adaptability measures than Monitoring scores. Our results indicate that independent processes underlying individual differences in interoceptive sensibility and emotional conceptualization contribute to emotion experiencing.

## Introduction

Traditional theoretical approaches posit that the perception and experience of a particular emotion depend on neural circuitries specialized in generating discrete affective responses (Adolphs and Anderson, 2018; Dolcos et al., 2020b). However, more recent perspectives, such as the theory of constructed emotions (TCE; Barrett and Lisa Feldman, 2006, Barrett, 2017a, b; Lindquist et al., 2012; MacCormack and Lindquist, 2017), suggest that the experience of an emotion results from the interaction of more general components that are not specific to emotion generation and whose final goal is to maintain the homeostasis of the organism (Barrett, 2017a, b). This view resembles neuroscientific models in suggesting that psychological events are the product of the interaction of large-scale networks (Deco et al., 2011; Lindquist et al., 2012; Barrett and Satpute, 2013; Wilson-Mendenhall et al., 2013; Kleckner et al., 2017). In the TCE, Barrett and colleagues assume that at least four components may be involved in the construction and experience of emotions, namely, core affect, conceptualization, attention, and the verbalization of emotions (Barrett and Lisa Feldman, 2006, Barrett, 2017a, b; Lindquist et al., 2012; MacCormack and Lindquist, 2017). In the current study, we focused on how potential correlates of core affect and conceptualization moderate the experience of emotions (for detailed reviews on attention and emotional verbalization see Barrett et al., 2004; Lindquist, 2017; Hoemann et al., 2019; Satpute and Lindquist, 2021).

Core affect refers to the mental representation of bodily changes that are sometimes, but not always, associated with pleasure or displeasure and arousal (Barrett and Russell, 1999; Lindquist et al., 2012; Barrett and Simmons, 2015; Barrett, 2017a; MacCornmack and Lindquist, 2017). Bodily changes are crucial to regulating energy expenditure and maintaining physiological, immunological, and hormonal equilibrium. As an active entity, our brain generates models that predict what the upcoming optimal bodily state should be in order to efficiently distribute and organize energy (Seth, 2013; Barrett and Simmons, 2015; Ainley et al., 2016). When afferent signals do not match with the expected optimal internal state, signals from the body are fed back to the brain as prediction errors to adapt to the current circumstances by reducing this mismatch (Barrett and Simmons, 2015). Core affect is, thus, directly influenced by interoceptive signals that are sent from the body to the brain.

Of note, it has been suggested that interoception comprises three distinct facets depending on the nature of the measurement: accuracy, sensibility, and awareness (Garfinkel et al., 2015; Critchley and Garfinkel, 2017). Interoceptive accuracy is understood as the objective accuracy in detecting internal bodily sensations (e.g., heart rate, respiration rate, stomach dilatation) and is typically measured using standard and objective behavioral tasks such as Heartbeat counting task or Whitehead heartbeat detection task (Critchley and Garfinkel, 2017; Smith et al., 2020; Legrand et al., 2021). Interoceptive sensibility refers to the subjective perception and beliefs about the internal focus and/or accuracy of an individual in perceiving interoceptive signals. Interoceptive sensibility is commonly assessed *via* self-report measures asking participants to make explicit propositional statements about how (in)accurately they perceive their bodily sensations, or how attentive they are to them (Brewer et al., 2016; Cabrera et al., 2018; Murphy et al., 2020; Gabriele et al., 2020). Interoceptive awareness, as the third interoceptive facet, reflects the meta-cognitive awareness of interoceptive accuracy, which is the degree of convergence between interoceptive accuracy and sensibility (Critchley and Garfinkel, 2017). Given the tight link between interoception and core affect, individual differences in interoceptive processing, especially interoceptive accuracy, could be considered a reliable index of core affect (Kleckner et al., 2017).

Interoceptive sensations and core affect *per se* do not construct an instance of emotion. They need to be categorized and made meaningful. This conceptualization process, the second component of emotion construction, occurs when the brain uses prior knowledge and experiences to give meaning to the bodily sensations felt in a particular moment within a particular context (Wilson-Mendenhall et al., 2011; Barrett, 2017a, b). Categorizing internal and/or external inputs thus allows to identify bodily sensations as meaningful entities and assign them causation. For instance, a pounding heart could be categorized as happiness in the context of meeting a romantic interest, or as exhaustion in the context of a race. Some common measures used to evaluate conceptualization are based on self-report. These questionnaires assess the beliefs of participants regarding their ability to mentally represent emotions (also known as emotional intelligence, awareness, or expertise) by asking them to evaluate how accurately they experience their emotions, or how attentive they are to them (Bagby et al., 1994; Swinkels and Giuliano, 1995; Kang and Shaver, 2004; see also Lindquist and Barrett, 2008; MacCormack and Lindquist, 2019; MacCormack et al., 2020, for a detailed review see Hoemann et al., 2020, for experimental manipulations of emotional conceptualization). Self-report measures of alexithymia, a sub-clinical condition characterized by a poor ability to identify and describe one’s own emotions, have also often been used to assess individual differences in emotional conceptualization (Lindquist and Barrett, 2008). Recent evidence further suggests that the integrity of the default mode network (DMN), as the potential primary network involved in conceptualization (Lindquist et al., 2012; Satpute and Lindquist, 2019), may constitute a neural correlate of this component (Liemburg et al., 2012; Imperatori et al., 2016; Takeuchi et al., 2016).

Previous studies investigating how individual differences in interoception and emotional conceptualization may relate to emotional experience focused on three main aspects: emotional intensity, activation or arousal, and granularity. Emotional intensity is defined as the strength with which a particular emotion is felt, ranging from high (e.g., “extremely happy”) to low (e.g., “not happy at all”). Emotional activation or arousal is a more general term encompassing the degree of activation in a specific situation and typically ranges from calm to active or excited (e.g., Lang et al., 1990; Cacioppo and Berntson, 1994; Reisenzein, 1994; Barrett and Russell, 1999; Kuppens et al., 2013). Although emotional intensity and arousal may overlap, emotional arousal is not always associated with high intensity, for instance, emotions such as satisfaction or sadness can be experienced with high intensity under low arousal states (Kuppens et al., 2013). Emotional granularity is defined as the ability to precisely differentiate emotions. People with high emotional granularity are able to label their emotional experience in precise terms (i.e., distinguishing between experiencing “sadness” and “compassion”) whereas those with low emotional granularity tend to use the same terms to describe different experiences (i.e., differentiating only between feeling “good” or “bad”; Lindquist and Barrett, 2008).

In one of the first studies investigating the relationship between interoceptive accuracy and emotional experience, Pollatos et al. (2007) observed that participants with high relative to low interoceptive accuracy experienced the viewing of unpleasant and pleasant scenes as more arousing, as indicated by higher subjective arousal ratings (see also, Wiens et al., 2000; Barrett et al., 2004; Critchley et al., 2004; Pollatos et al., 2005; Herbert et al., 2007, 2010; Pollatos and Schandry, 2008). Importantly, not only interoceptive processing but also individual differences in emotional conceptualization, seem to play a role in the intensity and activation of experienced emotions. For instance, Mantani et al. (2005) observed that imagined past emotional events were experienced with lesser intensity by participants with high, compared to low, alexithymia scores (see also, Stone and Nielson, 2001; Luminet et al., 2004). Similarly, Pollatos and Schandry (2008) observed that participants with high alexithymia scores rated emotional pictures as less arousing than those scoring low on this scale. Despite previous evidence linking interoceptive processing and emotional conceptualization to emotional intensity and arousal, little is known about how these constructs relate to emotional granularity. Although a positive association between individual differences in emotional conceptualization and emotional granularity has been theorized (Lindquist and Barrett, 2014; Smith et al., 2019), this question remains under-examined (Lindquist, 2013; Lindquist and Barrett, 2014; Erbas et al., 2016).

To shed more light on the role of interoception and emotional conceptualization in emotion experience, the current study aimed to investigate how individual differences in these constructs interact to moderate emotional intensity, arousal, and granularity. Unlike previous studies in which interoceptive processing was operationalized using objective measures (i.e., interoceptive accuracy), here, we used self-report measures of interoception, particularly focusing on interoceptive sensibility. Similarly, emotional conceptualization was evaluated using self-report measures. Emotional intensity, arousal, and granularity were extracted from two emotion experience tasks that involved standardized material (emotion differentiation task; ED, e.g., Nook et al., 2018) and self-experienced episodes (DRM; Barrett et al., 2001; Lee et al., 2017).

Based on previous literature, we expected that measures of interoception would show a positive relationship with emotional intensity and arousal, whereas measures of emotional conceptualization would show a positive association with emotional intensity, arousal, and granularity (Pollatos et al., 2007; Lindquist and Barrett, 2014).

Finally, higher interoceptive sensibility and emotional conceptualization scores are considered to reflect a more efficient functioning of the underlying components, leading to a better adaptation to the environment, and in turn, higher well-being (Ainley et al., 2016; Barrett et al., 2016; Khalsa et al., 2018; Hoemann et al., 2020). To test for that, we further examined the association between individual differences in interoceptive sensibility and emotional conceptualization and subjective reports of adaptability and well-being.

## Materials and Methods

### Participants

A total of 157 participants (135 women, 22 men; *M* age = 25.92; *SD* age = 8.39) took part in the two-session, online study in exchange for course credits. Each individual provided informed consent in accordance with the data protection laws of the University of Potsdam. Twenty-three participants were excluded from analysis because they reported one or more of the following excluding criteria: German proficiency level lower than C1 (i.e., advanced level), history of neurological disorder, undergoing psychological treatment at the moment of the study or having suffered any psychological disorder during the last year, and undergoing acute or long-term psychiatric treatment. In addition, participants were excluded based on their speed of completion as the online platform *soscisurvey.de* (Leiner, 2019a) calculates two indices of suspicious survey completion (Leiner, 2019b). The index *DEG_TIME* marks those participants who complete the survey exceptionally quickly relative to the rest of the sample. It is recommended to exclude individuals with scores larger than 100. The index *TIME_RSI* corresponds to the relative speed index and calculates the relative time to complete the questionnaire in comparison to the median of the overall sample (Leiner, 2019b). It is recommended that individuals with scores larger than 2, indicating that the questionnaire was completed in less than half the time required by the typical responder, are excluded. The final sample consisted of 131 participants (112 women, 19 men; mean age = 26.18).

### Questionnaires^1^

A series of questionnaires were selected to measure individual differences in interoception and conceptualization along with psychological well-being and adaptability.

### Interoception Scales

Although there are different questionnaires available that measure individual differences in interoceptive sensibility, they tend to focus on different aspects of interoception (e.g., physiological sensibility vs. self-regulation). Indeed, these questionnaires correlate weakly, suggesting they might be measuring different sub-constructs of interoceptive sensibility (see Desmedt et al., 2021). To address this heterogeneity, new questionnaires providing a clearer differentiation of facets of interoception have recently been developed (Brewer et al., 2016; Gabriele et al., 2020; Murphy et al., 2020). Because core affect relies on the ability to accurately perceive interoceptive signals, we chose to focus on questionnaires measuring physiological sensibility. We used recently developed questionnaires that assess this facet (i.e., beliefs of an individual concerning the (in)ability to perceive or differentiate physiological changes) along with scales that evaluate other facets of interoception.

#### Interoceptive Confusion Questionnaire (ICQ)

The Interoceptive Confusion Questionnaire (ICQ; Brewer et al., 2016) is a 20-item scale that evaluates the degree to which individuals have difficulties interpreting their own non-affective interoceptive states, such as hunger, muscle pain, or arousal (e.g., *I often find that I’m suddenly very thirsty; I only realize I am stressed when others tell me*). Responses are given on a 5-point Likert scale ranging from 1 (does not describe me) to 5 (describes me very well). The final score of the ICQ is the sum of all the items.

In the current study, we used a German version of the ICQ that is used for validation of other interoceptive questionnaires^3^. Similar to the original validation sample (Brewer et al., 2016), in the current study, the consistency of the ICQ was rather poor (Cronbach’s α = 0.55), however, we decided to use this scale because of its established construct validity (Brewer et al., 2016).

#### Interoceptive Accuracy Scale (IAS)

The interoceptive accuracy scale (IAS; Murphy et al., 2020) is a 21-item questionnaire that assesses the global beliefs concerning the ability of an individual to accurately perceive interoceptive signals (e.g., *I can always accurately perceive when my heart is beating fast; I can always accurately perceive when I am thirsty*). The items are answered using a 5-point Likert scale ranging from 1 (Disagree Strongly) to 5 (Agree Strongly). Total score of the IAS is calculated by summing all the items. In contrast to the ICQ, the IAS has shown good psychometric properties (Murphy et al., 2020). The IAS has been validated in an English-speaking sample, providing good construct and external validity, along with notable test-retest reliability and consistency (Murphy et al., 2020). Although the German validation is still in progress, our unpublished findings replicate the results from the original English version (see text footnote 2). In the current sample, the IAS showed good consistency (Cronbach’s α = 0.84).

#### Multidimensional Assessment of Interoceptive Awareness Version-2

The Multidimensional Assessment of Interoceptive Awareness Version-2 (MAIA-2; Mehling et al., 2018) consists of 37 items divided into 8 scales, and measuring multiple dimensions of interoception, including Noticing (4 items; e.g., *I notice when I am uncomfortable in my body*), Not-Distracting (6 items; *I distract myself from sensations of discomfort*), Not-Worrying (5 items; e.g., *When I am in discomfort or pain I can’t get it out of my mind*), Attention Regulation (7 items; e.g., *I can return awareness to my body if I am distracted*), Emotional Awareness (5 items; e.g., *I notice that my breathing becomes free and easy when I feel comfortable)*, Self-Regulation (4 items; e.g., *I can use my breath to reduce tension*), Body Listening (3 items; e.g., *I listen to my body to inform me about what to do*), and Trust (3 items; e.g., *I trust my body sensations*). The MAIA-2 aims to differentiate between adaptive and maladaptive styles of interoception, related to resilience and anxiety, respectively (Mehling et al., 2018; Reis, 2019). Each item is rated on a 6-point Likert-scale, ranging from 0 (never) to 5 (always). Scores for each scale are calculated by performing the average of the corresponding items. In the current sample, the Cronbach’s α indices of the MAIA-2 subscales range from 0.5 to 0.87.

### Conceptualization Scales

Because the conceptualization component is involved in the categorization of emotions during a particular event, it is expected that a more efficient conceptualization is reflected by higher accuracy in perceiving and understanding emotions. To evaluate individual differences in conceptualization, we selected a series of questionnaires that assess how (in)accurately one perceives their own emotions (Bagby et al., 1994; Swinkels and Giuliano, 1995; Kang and Shaver, 2004).

#### Toronto Alexithymia Scale (TAS-20)

The Toronto Alexithymia Scale (TAS-20; Bagby et al., 1994) consists of 20 items grouped in three subscales: Difficulties Identifying Feelings (7 items; e.g., *I am confused about what emotion I am feeling)*, Difficulties Describing Feelings (5 items; e.g., *It is difficult for me to find the right words for my feelings*), and Externally Oriented Thinking (8 items; e.g., *I prefer talking to people about their daily activities rather than their feelings)*. Items are rated on a five-point Likert scale, ranging from 1 (does not describe me) to 5 (describes me). Previous studies showed that the TAS-20 has a good consistency and construct validity (Bagby et al., 1994). The total score of the TAS-20 is the sum of all the items. In the current sample, good consistency of the TAS-20 was observed (Cronbach’s α = 0.83).

#### Mood Awareness Scale (MAS)

The Mood Awareness Scale (MAS; Swinkels and Giuliano, 1995) consists of 10 items that evaluate the attention toward one’s mood states. The MAS is subdivided into two subscales: the mood labeling subscale (5 items; e.g., *Right now I know what kind of mood I’m in*) evaluates the ability to identify, categorize or give a name to feelings (Swinkels and Giuliano, 1995); the mood monitoring subscale (5 items; e.g., *I find myself thinking about my mood during the day*) assesses the degree of focus or vigilance on the affective states of an individual. Items are rated on a 6-point Likert-scale, ranging from 1 (disagree very much) to 6 (agree very much). The scores for each scale are calculated by summing the corresponding items, and the total score is calculated by summing over all the items. In the current sample, the MAS showed good consistency (Cronbach’s α: 0.70–0.79).

#### Range and Differentiation of Emotional Experience Scale (RDEES)

The Range and Differentiation of Emotional Experience Scale (RDEES; Kang and Shaver, 2004) consists of 14 items and two subscales, the Differentiation scale (7 items; e.g., *I am aware of the subtle differences between the feelings I have*), and the Range scale (7 items; e.g., *I experience a wide range of emotion*). The ratings are given using a 5-point Likert-scale ranging from 1 (it does not describe me very well) to 5 (describes me very well). The score for each scale is calculated by summing the corresponding items. The sum of all items forms the total RDEES score. In the current sample, the RDEES showed good consistency (Cronbach’s α: Range = 0.75, Differentiation = 0.8, Total = 0.81).

#### Trait Meta-Mood Scale (TMMS)

The Trait Meta-Mood Scale (TMMS; Salovey et al., 1995) is a 30-item questionnaire that evaluates one’s abilities to manage and reflect upon emotions. The TMMS is divided into three subscales, the attention subscale (13 items; e.g., *I pay a lot of attention to how I feel*) which measures the attention devoted to the feelings of an individual, the clarity subscale (11 items; e.g., *I usually know my feelings about a matter*) which assesses the clarity of the experienced feelings, and the repair subscale (6 items; e.g., *When I become upset, I remind myself of all the pleasures in life*) which evaluates the beliefs about ending negative mood states or prolonging positive ones. The items are rated on a 5-point Likert-scale ranging from 1 (strongly disagree) to 5 (strongly agree). The Cronbach’s α denoted good consistency (Cronbach’s α:0.8–0.87).

### Well-Being Scale

#### Well-Being Questionnaire (W-BQ12)

The Well-Being Questionnaire (W-BQ12; Mitchell and Bradley, 2001) consists of 12 items, evaluating psychological well-being by asking about the frequency of experiencing different feelings over the past few weeks. Each item is scored using a 4-point Likert-scale, ranging from 0 (not at all) to 3 (all the time). The W-BQ12 is divided into three 4-item subscales: Negative Well-Being (NWB; e.g., *I have crying spells or feel like it*), Positive Well-Being (PWB; e.g., *I have lived the kind of life I wanted to*) and Energy (e.g., *I feel energetic, active, or vigorous*). The scores for each subscale are calculated by summing the scores of each item. The general well-being score is calculated using the following formula: 12-NWB + Energy + PWB. In our sample, the W-BQ12 showed poor consistency (Cronbach’s α = 0.5). However, we decided to use this scale due to its established construct validity.

### Tasks

Emotional experience was induced by way of two different tasks, allowing us to measure emotional intensity, arousal, and granularity scores (see Analysis section).

#### Emotion Differentiation Task

**FIGURE 1:**
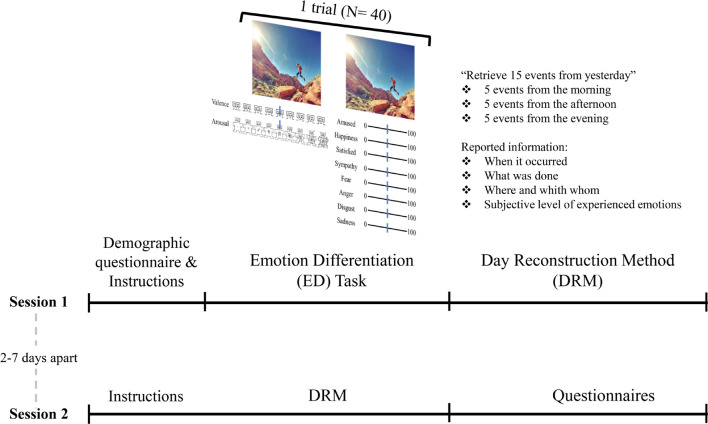
Visual representation of the procedure. In the first of the two-session study, participants performed the Emotion Differentiation (ED) task (if they chose to) and the first part of the Day Reconstruction Method (DRM). In session 2, which took place 2 to 7 days after session 1, participants completed the second part of the DRM and the questionnaires. Picture source: https://pxhere.com/.

The Emotion Differentiation (ED) task is an online adaptation of previous laboratory-based protocols (Nook et al., 2018; Israelashvili et al., 2019). This task is designed to assess how participants identify the experienced emotions which are evoked by a series of scenes. A total of 40 pictures (20 negative and 20 positive) extracted from the *International Affective Picture System* (IAPS; Lang et al., 2008) were used to evoke emotions. Images were chosen to represent a heterogeneous pool of scenes with different content, valence, and arousal levels. The normative valence and arousal ratings of the selected images were as follows: valence = 7.04, arousal = 4.86, for pleasant images, and valence = 3.02, arousal = 5.59, for unpleasant images. Each image was presented twice consecutively (see Figure 1). In the first presentation, participants were asked to rate their experienced level of valence and arousal in response to the picture, using a sliding bar superimposed over a miniature representation of the Self-Assessment Manikin Scale (SAM; Lang et al., 2008). The position of the sliding bar was then quantified as a percentage of the scale (i.e., distance between the left-most point and the rating of the scale). In the second presentation, participants were instructed to indicate to what extent they felt each of the following eight emotions: amusement, happiness, satisfaction, sympathy, fear, anger, disgust, and sadness. To give their ratings, participants could move a sliding bar along the scale that ranged from 0 (not at all) to 100 (very much). The initial position of the sliding bar was always in the middle (50). The presentation of each of the 40 images was fully randomized. Although there was no time limit for rating each picture, participants were instructed not to overthink their responses.

#### Day Reconstruction Method

As the second emotional task, we used an online-adapted version of the DRM (Barrett et al., 2001; Lee et al., 2017). The DRM was conducted two times on two different days. On each day, participants were asked to recall up to 15 episodes that happened to them the previous day (5 from the previous morning, 5 from the afternoon, and 5 from the evening), leading to up to 30 episodes. For each episode, participants were asked to report when it occurred, what they were doing, where and with whom they were, and the level to which they experienced the following positive and negative emotions: amusement, awe, contentment, excitement, gratitude, happiness, love, pleasure, pride, serenity, anger, boredom, disgust, dissatisfaction, downheartedness, embarrassment, fear, sadness, and fatigue. The responses here were given on a 7-point Likert-scale, ranging from 0 (not at all) to 6 (very much).

### Procedure

Each of the two sessions of the study lasted for about 45 min. In the first session, participants first completed a demographic questionnaire. Thereafter, the description of the ED task was provided. After being informed that the ED task contained very explicit scenes (e.g., mutilations or sex-related contents), participants could choose whether to perform the ED task or not. If they did decide to perform the ED task, the instructions for the task were presented and, after two practice trials, the main task was conducted. Thereafter, the instructions for the DRM were presented followed by the task. If participants decided not to take part in the ED task, they were immediately directed to the DRM task.

Between 2 and 7 days after the first session, participants were invited to take part in the second session. This session began with the DRM followed by the self-report questionnaires. The questionnaires were administered in two fixed orders which were counterbalanced across participants. No questionnaires or tasks other than those reported in this section were administered.

### Analysis

#### Association Between Interoceptive Sensibility and Conceptualization Scores

To investigate the relationship between interoception and emotional conceptualization questionnaires, Pearson’s correlation analyses were performed. Thereafter, Principal Component Analysis (PCA) was performed to extract the factors underlying the questionnaire scores. Initially, a PCA with varimax rotation (i.e., to maximize simple structure) was performed to identify the number of relevant underlying factors.

Of note, The MAIA-2 is a heterogeneous questionnaire that not only assesses the ability to perceive and “listen to” the physiological changes of an individual (i.e., Noticing, Emotional Awareness, Trusting, Body Listening, Attention Regulation scales), but also adaptive regulatory strategies when dealing with interoceptive changes (i.e., Non-Distracting, Not-Worrying, Self-Regulation scales). Reflecting this heterogeneity, scores of the MAIA-2 have been considered as an indicator of interoceptive sensibility along with a correlate for maladaptive or beneficial interoceptive strategies (Mehling et al., 2018). Similarly, scores of the MAIA-2 have been shown to predict psychological improvement trajectories, and are negatively related to several mental health symptoms and emotion regulation difficulties (Barker, 2019; Eggart and Valdés-Stauber, 2021; Millon and Shors, 2021). Considering the different constructs that the MAIA-2 comprises with each subscale, we decided to include the subscales related to physiological sensibility, namely, the Noticing, Emotional Awareness, Trusting, Body Listening, and Attention Regulation subscales, in the PCA. The subscales that assess the usage of maladaptive or beneficial interoceptive strategies, namely, the Non-Distracting, Not-Worrying, and Self-Regulation subscales, were used as indices of adaptability.

#### Extraction of Emotional Intensity, Arousal and Granularity Indexes

Emotional intensity was taken as the intensity of the emotional word with the highest rating for each trial, which was averaged across trials. Emotional arousal was extracted by averaging the experienced arousal across trials in the ED task. Because intensity and arousal scores were negatively skewed (*skewness* > −0.79), they were normalized using the following formula: *sqrt(max(S* + *1) – S)*, where *S* refers to the mean intensity/arousal scores. Normal distribution was achieved after applying this transformation (0.06 < *skewness* < 0.09).

Emotional granularity was extracted from the ED and DRM tasks by computing the intra-class correlation index (ICC; Kalokerinos et al., 2019) for positive and negative emotional adjectives or nouns separately, resulting in two ICC indices per participant, one ICC for positive and one for negative emotions. The ICC was computed using the package *irr*^4^. Both participants and emotion words were considered as random effects (i.e., two-way model) and the unit was set to average (see also Kalokerinos et al., 2019). Higher ICC scores indicate that the ratings for different emotion types are highly correlated. On the other hand, a lower ICC is indicative of a lower correlation between emotion ratings. It is assumed that participants with higher ICC experience emotions in a similar fashion across trials, whereas participants with lower ICC experience each emotion independently, and thus, are able to distinguish emotions in a detailed manner. As reliable ICC scores range between 0 and 1, participants with negative, uninterpretable ICCs were excluded (12 participants for negative and 1 for positive adjectives in the DRM, and 1 participant for negative adjectives in the ED task; Kalokerinos et al., 2019). We normalized the ICC scores using Fischer’s transformation (Kalokerinos et al., 2019). The ICC scores were reversed (−1 × ICC) to make higher values correspond to higher granularity. The relation between emotional intensity, arousal, and granularity was tested using Pearson’s correlations.

#### Association Between Principal Component Analysis Components and Emotional Experience

We used multiple regression analyses to investigate the relationship between the factor scores extracted from the interoception and emotional conceptualization questionnaires and emotional intensity, arousal, and granularity extracted from the ED and DRM tasks. For this purpose, we used the factor variables as predictors and the emotional experience scores as predicted variables. For the scores from the DRM task, we also added the number of retrieved episodes as a predictor to control for differences in the number of retrieved episodes.

#### Association Between Principal Component Analysis Factors and Well-Being and Adaptability

Finally, we used correlational analysis to investigate the relationship between the factor scores and indices of adaptability and well-being. Correlations between the factor scores and well-being and adaptability indices were compared with the Pearson and Filon’s Z, using the *cocor* package in R (Diedenhofen and Musch, 2015).

## Results

### Correlation Analysis

Table 1 contains the correlational analysis between all questionnaire scales from a total of 109 (83% of the included sample) participants.

**TABLE 1:** Pearson’s correlation matrix for interoception and conceptualization scales.

*Questionnaires*	*Variable*	*ICQ*	*IAS*	*Noticing*	*Attn Reg*	*Emo Awr*	*Body list*	*Trust*	*Desc feel*	*Id feel*	*Extern think*	*Labeling*	*Monitoring*	*Range*	*Diff*	*Clarity*	*Attention*
*IAS*	*IAS*	−0.52***	−														
*MAIA-2*	*Noticing*	−0.44***	0.44***	−													
	*Attn Reg*	−0.43***	0.34***	0.49***	−												
	*Emo Awr*	−0.39***	0.30**	0.56***	0.41***	−											
	*Body List*	−0.37***	*0*.21*	0.31***	0.43***	0.52***	−										
	*Trust*	−0.46***	0.26**	0.33***	0.55***	0.41***	0.48***	−									
*TAS-20*	*Desc Feel*	0.31**	−0.22*	–0.15	−0.40***	−0.29**	−0.37***	−0.31**	−								
	*Id Feel*	0.55***	−0.55***	−0.41***	−0.49***	−0.27**	−0.41***	−0.53***	0.52***	−							
	*Ext Think*	0.17	−0.27**	–0.07	−0.26**	−0.25**	−0.30***	−0.28**	0.37***	0.37***	−						
*MAS*	*Labeling*	−0.44***	0.43***	0.36***	0.50***	0.26**	0.43***	0.37***	−0.71***	−0.78***	−0.42***	−					
	*Monitoring*	–0.16	0.16	*0*.21*	0.48**	0.34***	0.34***	0.13	–0.14	–0.16	−0.20*	0.15	−				
*RDEES*	*Range*	−0.20*	0.19**	0.02	0.18	0.12	0.12	0.03	−0.24*	–0.16	−0.26**	0.28**	0.31**	−			
	*Diff*	−0.32***	0.43***	0.28**	0.29**	0.27**	0.27**	0.15	−0.39***	−0.44***	−0.32***	0.49***	0.31**	0.38***	−		
*TMMS*	*Clarity*	−0.51***	0.49***	0.34***	0.50***	0.27**	0.53***	0.44***	−0.53***	−0.77***	−0.27***	0.77***	0.20*	0.25**	0.49***	−	
	*Attention*	−0.20*	0.18	0.26**	0.27**	0.37***	0.39***	*0*.20*	−0.23*	–0.17	−0.37***	0.29**	0.52***	0.28**	0.28**	0.32***	−
	*Repair*	−0.29**	0.18	0.36***	0.37***	0.38***	0.29**	0.53***	−0.28**	−0.43***	–0.18	0.35***	0.15	0.08	*0*.20*	0.38***	0.28**

*p < 0.05, **p < 0.01, ***p < 0.001. Attn. Reg.: Attention Regulation; Emo Awr: Emotion Awareness; Body List: Body Listening; Desc Feel: Describing Feelings; Id Feel: Identifying Feelings; Ext Think: Externalizing Thinking; Diff: Differentiation.

### Principal Component Analysis (PCA)

An initial PCA with rotation varimax revealed that four factors with eigenvalues larger than 1 explained a total of the 65.1% of the variance (Factor 1: eigenvalue = 6.60, percentage of variance explained: 38.9; Factor 2, eigenvalue = 1.71, percentage of variance explained: 10; Factor 3, eigenvalue = 1.54, percentage of variance explained: 7.54; Factor 4, eigenvalue = 1.21, percentage of variance explained: 7.1; See Table 2). However, some of the factors were mostly loaded by subscales from the same questionnaire (e.g., Factor 1 by subscales of the TAS-20; Factor 2 by subscales of the MAIA-2). To ensure that the extracted components reflected general constructs underlying all the variables, we decided to force the PCA to two factors (see Table 2). In factor 1, ICQ and the subscales from TAS-20 loaded negatively, whereas IAS, the subscales Attention Regulation and Trusting of the MAIA-2, MAS Labeling, RDEES Differentiation, TMMS Clarity, and TMMS Repair loaded positively. In factor 2, the subscales Noticing and Emotional Awareness of the MAIA-2, MAS Monitoring, RDEES Range, and TMMS Attention loaded positively. The subscale Body Listening loaded in both factors equally.

**TABLE 2:** Principal Component Analysis (PCA) on interoception and emotional conceptualization scales.

PCA with factors eigenvalue > 1						PCA forced to 2 Factors	
*Questionnaires*		F1	F2	F3	F4	F1: *Sensibility*	F2: *Monitoring*
*ICQ*	*ICQ*		–0.375	**−0.654**		**−0.631**	
*IAS*	*IAS*			**0.786**		**0.581**	
*MAIA-2*	*Noticing*			**0.651**	0.375	0.379	**0.547**
	*Attn Reg*		**0.649**	0.314		**0.632**	
	*Emo Awr*		0.520		**0.587**		**0.718**
	*Body List*	0.328	**0.575**		0.360	*0.473*	*0.473*
	*Trust*		**0.790**			**0.613**	
*TAS-20*	*Decs Feel*	**−0.752**				**−0.669**	
	*Id Feel*	**−0.585**	–0.412	**−0.539**		**−0.892**	
	*Ext Think*	**−0.622**		0.388		**−0.386**	–0.312
*MAS*	*Labeling*	**0.771**				**0.854**	
	*Monitoring*				**0.822**		**0.839**
*RDDES*	*Range*	0.397	–0.321		**0.492**		**0.431**
	*Diff*	0.455		**0.523**	0.343	**0.474**	0.380
*TMMS*	*Clarity*	**0.627**	0.355	0.475		**0.836**	
	*Attention*				**0.713**		**0.721**
	*Repair*		**0.639**		–0.319	**0.471**	

*Attn. Reg.: Attention Regulation; Emo Awr: Emotion Awareness; Body List: Body Listening; Desc Feel: Describing Feelings; Id Feel: Identifying Feelings; Ext Think: Externalizing Thinking; Diff: Differentiation.*

The factor scores did not differentiate between interoceptive sensibility and emotional conceptualization scales. Instead, they revealed overlapping variance between measures of both components. Factor 1 mostly comprised scales measuring sensibility toward perceiving physiological changes and emotion and was named “*Sensibility.”* Factor 2 consisted of scales that are related to perceptions about attentional resources devoted to physiological and emotional aspects and was labeled “*Monitoring*.” Table 2 shows the loading scores from each of the scales.

### Relation Between Principal Component Analysis Factors and Emotional Intensity and Granularity

#### Emotion Differentiation (ED) Task

A total of 127 participants (96% of the included sample) performed the ED task (Table 3). Correlational analysis between emotional intensity, arousal, and granularity scores showed that emotional intensity correlated positively with arousal, *r*(126) = 0.25, *p* = 0.006, and with emotional granularity for negative words, *r*(125) = 0.28, *p* < 0.001. However, no significant association was found with emotional granularity for positive words, *r*(126) = 0.06, *p* = 0.45. Arousal scores showed no significant association with emotional granularity for positive [*r*(126) = −0.01, *p* < 0.89] or negative words [*r*(125) = 0.084, *p* = 35], whereas emotional granularity for positive words correlated positively with emotional granularity for negative words, *r*(125) = 0.25, *p* = 0.004.

**TABLE 3:** The descriptive statistics for the Emotion Differentiation (ED) task and the Day Reconstruction Method (DRM) for emotional arousal, intensity, and granularity scores.

	Emotion differentiation (ED) task				Day reconstruction method (DRM)		
	*Granularity pleasant*	*Granularity unpleasant*	*Emotional intensity*	*Emotional arousal*	*Granularity pleasant*	*Granularity unpleasant*	*Emotional intensity*
Valid	127	126	127	127	129	118	130
Missing	0	1	0	0	1	12	0
Mean	–1.975	–1.005	4.183	4.263	0.193	0.462	1.440
Std. Deviation	0.386	0.272	1.115	1.286	0.125	0.218	0.200
Skewness	–0.297	–0.017	–0.058	0.067	1.618	0.442	0.085
Std. Error of Skewness	0.215	0.216	0.215	0.215	0.213	0.223	0.212
Minimum	–3.042	–1.645	1.000	1.000	0.026	0.090	1.000
Maximum	–1.003	–0.293	7.180	7.458	0.798	0.974	1.959

##### Sensibility and Monitoring did not predict either emotional intensity, arousal, or granularity

Multiple regression analysis revealed no association between the factor scores and emotional intensity: Monitoring: *t*(84) = 0.70, *p* = 0.48, β = 0.076; Sensibility: *t*(84) = 0.83, *p* = 0.41, β = 0.091; Monitoring × Sensibility: *t*(84) = 0.1, *p* = 0.91, β = −0.07. Similarly, no association was observed between the factor scores and mean arousal scores: Monitoring: *t*(84) = 0.21, *p* = 0.84, β = 0.02; Sensibility: *t*(84) = 0.13, *p* = 0.89, β = 0.012; Monitoring × Sensibility: *t*(84) = 0.44, *p* = 0.66, β = 0.048.

Neither granularity scores for positive nor negative emotions showed a significant association with the factor scores. For positive emotions: Monitoring: *t*(84) = −1.0, *p* = 0.31, β = −0.109; Sensibility: *t*(84) = −0.50, *p* = 0.61, β = −0.056; Monitoring × Sensibility: *t*(84) = −0.05, *p* = 0.96, β = −0.01. For negative emotions: Monitoring: *t*(84) = −0.5, *p* = 0.61, β = −0.054; Sensibility: *t*(84) = 0.45, *p* = 0.65, β = 0.05; Monitoring × Sensibility: *t*(84) = 0.89, *p* = 0.39, β = 0.09.

#### Day Reconstruction Method (DRM)

A total of 130 participants (99% of the included sample) performed the DRM (Table 3). Correlational analysis between emotional intensity and granularity scores showed that emotional intensity did not correlate with emotional granularity for positive [*r*(128) = 0.13, *p* = 0.13] or negative words [*r*(117) = −0.03, *p* = 0.74]. Additionally, no association was observed between emotional granularity for positive and negative words, *r*(116) = 0.13, *p* = 0.17.

##### Sensibility and Monitoring predict lower emotional intensity

**FIGURE 2:**
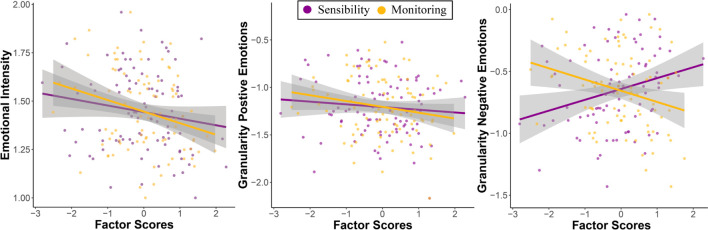
Association between emotional intensity and granularity scores and Sensibility and Monitoring factor scores in the DRM.

Multiple regressions indicated that Monitoring significantly predicted lower emotional intensity *t*(86) = 3.056, *p* = 0.003, β = −0.31. Sensibility was associated with emotional intensity at a trend level, *t*(86) = −1.98, *p* = 0.05, β = −0.20. No significant interaction between factor scores was observed, *t*(86) = −1.54, *p* = 0.12, β = 0.15. The number of events reported was also related to emotional intensity at a trend level *t*(86) = 1.75, *p* = 0.082, β = −0.17 (Figure 2).

##### Differential effects of sensibility and monitoring on emotional granularity

Granularity scores for positive emotions were negatively associated with Monitoring, *t*(86) = −1.99, *p* = 0.049, β = −0.21 but not with Sensibility, *t*(86) = −1.17, *p* = 0.24, β = −0.120. No significant interaction effects were observed, *t*(86) = −1.07, *p* = 0.28, β = −0.11 (Figure 2).

Granularity scores for negative emotions were differently moderated by Monitoring and Sensibility. Whereas Monitoring predicted lower granularity scores, *t*(78) = −2.96, *p* = 0.004, β = −0.31, Sensibility was associated with higher granularity, *t*(78) = 2.59, *p* = 0.011, β = 0.27. No interaction effects were observed, *t*(78) = 0.07, *p* = 0.94, β = 0.001 (Figure 2).

### Association Between Principal Component Analysis Factors and Adaptability and Well-Being Scales

Table 4 shows the correlation of the Sensibility and Monitoring factors with the adaptability (MAIA-2) and well-being (W-BQ12) scales, and Z scores for the comparison of the correlations.

**TABLE 4:** Pearson’s correlation between factor scores and well-being and adaptability measures.

Factor and questionnaires	Variable	Sensibility	Monitoring	Z scores
	*Monitoring*	0.00	−	
*MAIA-2*	*not Distracting*	0.17	0.09	0.64
	*not Worrying*	0.40***	−0.20*	4.94***
	*self-Regulation*	0.54***	0.38***	1.40
*W-BQ12*	*Positive*	0.45***	0.24**	1.8
	*Negative*	−0.54***	0.07	−5.23***
	*Energy*	0.47***	0.11	2.93**
	*Total*	0.58***	0.12	3.94***

*p < 0.05, **p < 0.01, ***p < 0.001. Correlation indices were compared between factors (Z scores).

Although both factors showed significant correlations with the adaptability and well-being scales, in general, Sensibility showed larger correlations than Monitoring, indicating that Sensibility and Monitoring contribute differently to these scales.

## Discussion

In the current study, we aimed to investigate how individual differences in interoceptive sensibility and emotional conceptualization interact to moderate different facets of the emotional experience, namely, emotional intensity, arousal, and granularity. We observed that subjective measures of interoceptive sensibility were significantly correlated with measures of emotional conceptualization. PCA analysis revealed two independent factors, labeled Sensibility and Monitoring, in which measures of interoceptive sensibility and emotional conceptualization shared variance. The two factors had somewhat different effects on emotion experience, particularly in the DRM (but not in the ED) task. Sensibility was negatively (albeit non-significantly) related to emotional intensity and granularity for positive words, but positively related to granularity for negative words, whereas Monitoring was negatively related to emotional intensity and granularity for both positive and negative words. Additionally, the two factors showed differential associations with measures of well-being and adaptability: Sensibility scores were more strongly associated with greater well-being and adaptability measures than Monitoring scores.

### Association Between Interoceptive Sensibility and Emotional Conceptualization

We observed significant associations between self-report measures of interoceptive sensibility and emotional conceptualization. Specifically, self-report measures of interoceptive (in)accuracy were related to scales measuring (in)accuracy or clarity of detecting emotional states, convening in a common factor labeled Sensibility. Moreover, scales assessing how often attentional resources are deployed to bodily signals were related to a variety of self-report measures that assess the amount of attentional resources devoted to the emotions of an individual, overlapping in a factor labeled Monitoring.

The factor Sensibility reflects self-beliefs on how well one distinguishes, labels, and understands their physiological and emotional state. The convergence between self-beliefs of accuracy and/or confidence of two different entities is in line with recent findings, showing a moderate association between subjective (i.e., confidence ratings), but not objective accuracy scores of interoception and exteroception tasks (Legrand et al., 2021). Confidence about the accuracy of an individual in behavioral performance, and potentially, when detecting bodily changes and emotions, is an important aspect to guide adaptive behavior, particularly in the absence of feedback (Fleming and Daw, 2017). In this line, a positive association has been found between confidence and objective accuracy in various tasks (e.g., Yonelinas, 2002; Martino et al., 2013; Fleming and Daw, 2017; Murphy et al., 2020; Legrand et al., 2021).

The Monitoring factor reflects a general tendency to devote attentional resources to the internal physiological and emotional states of an individual. The role of selective and executive attention is crucial in the construction and experience of emotions (Barrett, 2017a; Smith et al., 2019). Which aspect of the ongoing processing the attention is deployed to, e.g., either to the bodily changes, or the surrounding environment, may have a strong influence on the interpretation of the current state of an individual (Barrett et al., 2004).

Previous theoretical models and empirical studies suggest that two different but complementary processes influence the disposition to understand and attend to physiological and emotional states (Boden and Thompson, 2017; Murphy et al., 2019). Our results support and extend this distinction by showing that these independent processes similarly relate to both physiological and emotional states. Within the framework of TCE, the Sensibility factor may be associated with individual differences in conceptualization, whereas Monitoring may be associated with individual differences in attentional processes. However, future studies that combine self-report measures with objective and/or physiological correlates are needed to provide more insights into the distinction between these components.

### Association Between Sensibility and Emotional Granularity, Well-Being, and Adaptability

Active inference accounts of emotion predict a positive association between the beliefs of an individual in understanding their own emotions and the ability to precisely use emotion concepts and differentiate between them (Lindquist and Barrett, 2014; Smith et al., 2019). In support of this assumption, we observed that Sensibility scores were positively related to emotional granularity for negative words. Thus, these results suggest that individual differences in conceptualization moderate the extent of differentiation between experienced negative emotions^5^.

According to the theory of constructed emotions (Barrett, 2017a, b), accurately identifying the actual internal state, either emotional or physiological, may activate more accurate predictions. This, in turn, can lead to better regulation of the available resources and help to prepare more adequate actions that favor the maintenance of homeostasis. For instance, if someone can accurately identify and differentiate between hunger or sadness, a series of more precise predictions may become accessible. These predictions would allow the person to act upon their needs or feelings and produce specific actions that lead to the ceasing of hunger or sadness, like getting some food or calling a close friend in search of support.

Importantly, this adaptive behavior may then result in greater psychological well-being and adaptability (Lindquist and Barrett, 2014). Correspondingly, the higher emotional granularity for negative words has been positively associated with healthy and adaptive behaviors such as the use and efficacy (Barrett et al., 2001; Kalokerinos et al., 2019) of emotional regulation strategies. Also, emotional granularity has been negatively related to depressive and social anxiety symptomatology, and it has been suggested as a correlate of resilience against the development of psychological disorders (Tugade et al., 2004; Kashdan et al., 2010; Demiralp et al., 2012; see also Erbas et al., 2014; Kashdan and Farmer, 2014). Here, we observed a positive association between Sensibility scores and well-being and adaptability scores, thereby providing further evidence for the association between correlates of conceptualization and well-being and adaptability.

### Association Between Monitoring and Emotional Intensity and Granularity

In the current study, we observed a negative relationship between Monitoring and granularity for negative and positive words. These findings indicate that participants with a higher tendency to attend to the internal state of an individual (i.e., physiological and/or emotional) showed a higher overlap between representations of emotional categories. Since lower differentiation between emotions implies that heterogeneous experiences are collapsed within the same emotional category, participants with lower granularity may have difficulties identifying the most appropriate set of predictions and actions to deal with different situations that they categorize within the same emotional label. In turn, they may require the engagement of more attentional resources to their current state to make a proper evaluation. However, this interpretation is merely speculative and requires future research, because, in the current study, we did not examine a causal relationship.

Monitoring scores were also negatively related to emotional intensity. This result indicates that a higher tendency to focus on the emotions of an individual was associated with lower experienced emotional intensity. Previous studies found that focusing on emotional aspects during the experience or retrieval of an emotional event increases the experienced emotional intensity and arousal, whereas focusing on non-emotional aspects of the event decreases the emotional intensity and arousal (Denkova et al., 2015; Iordan et al., 2019; Dolcos et al., 2020a, b). Based on that, the current findings suggest that participants with a higher tendency to focus on their emotions during the experience of an emotional episode, may invest their attentional resources in different aspects of the emotional event (i.e., what causes the emotion, what emotion is felt), reducing the experienced emotional intensity.

### Limitations and Future Considerations

In the current study, we did not observe any associations between Sensibility and Monitoring scores and the indexes of emotional experience from the ED task, which may be due to several reasons. Unlike the DRM, where participants idiosyncratically indicate how they felt in previously experienced events, the emotional events (i.e., images) in the ED task were pre-selected (standardized emotional pictures). Although these pictures were previously shown to modulate the extent of experienced valence and arousal, they may not evoke specific emotions. Another important aspect is that in the ED task, eight emotional labels (i.e., four positive and four negative) were used, whereas in the DRM, a total of 18 were provided. It could thus be that the eight available emotion labels did not sufficiently represent the evoked emotional state. Of note, in previous studies that successfully used the ED task, either more emotional labels or only single-valence words (i.e., negative) were used as anchors (Nook et al., 2018; Erbas et al., 2019; Israelashvili et al., 2019). This suggests that, when using standardized stimuli, a wider range of emotion labels is needed to ensure that the evoked emotions are represented in the provided labels.

In the current study, we assessed interoceptive processing using self-report measures. To gain more insights into the role of other facets of interoception in the emotional experience, future studies could use measures such as the Heartbeat counting task, the Whitehead heartbeat detection task, or heart-evoked potentials, which are more closely related to interoceptive accuracy (Critchley and Garfinkel, 2017).

Our sample primarily consisted of young adults and mainly featured female participants, which may constrain the generalizability of our results. In particular, considering that interoceptive sensibility scores and different aspects of the emotional experience may differ between genders and change across the life-span, future research is needed to clarify how these relationships are moderated by gender and aging (Grabauskaitë et al., 2017; Nook et al., 2018; MacCormack et al., 2021; Nook, 2021).

In summary, in the current study, we used self-report measures of interoception and emotional conceptualization to investigate how they interact in moderating different aspects of the emotional experience, namely, emotional intensity, arousal, and granularity. The interrelation between interoception and emotional conceptualization scales revealed two latent constructs that differently moderate the emotional experience. The Sensibility factor, which reflects beliefs of the accuracy of an individual in detecting internal (i.e., physiological and emotional) states, predicted higher granularity for negative words. The Monitoring factor, interpreted as the tendency to focus on the internal states of an individual, was negatively related to emotional granularity, intensity, and diminished psychological well-being. Additionally, the two factors showed differential associations with measures of well-being and adaptability. Sensibility scores were more strongly associated with greater well-being and adaptability than Monitoring scores. Thus, within inference accounts of emotion, these two factors could be interpreted as part of the intertwined components that contribute to the construction and experience of emotions.

## Significance Statement

It has been suggested that different psychological processes, including core affect (mental and neural representation of bodily changes) and conceptualization (meaning-making based on prior experiences and semantic knowledge), are involved in the formation of emotions. In the current study, we used self-report measures of interoceptive sensibility and emotional conceptualization (as potential correlates of these components) to investigate how they interact to moderate different aspects of the emotional experience, particularly emotional intensity, arousal, and granularity. The interrelation between interoceptive sensibility and emotional conceptualization scales revealed two latent constructs that differently moderate the emotional experience. The Sensibility factor, interpreted as a construct that reflects beliefs about the accuracy of an individual in detecting internal physiological and emotional states, predicted higher granularity for negative words. The Monitoring factor, interpreted as the tendency to focus on the internal states of an individual, was negatively related to emotional granularity and intensity. Additionally, the two factors showed differential associations with measures of well-being and adaptability. Particularly, Sensibility scores were more strongly associated with greater well-being and adaptability measures than Monitoring scores. These findings emphasize the role of these two constructs within the intertwined components that contribute to the construction and experience of emotions.

## Data Availability Statement

The raw data supporting the conclusions of this article will be made available by the authors, without undue reservation.

## Ethics Statement

Data from the current study are part of a larger project involving human participants that was reviewed and approved by the Ethics Committee of the University of Potsdam. The participants provided their written informed consent to participate in this study.

## Author Contributions

CV-B conceived the idea, defined the design, programmed and analyzed the data, and drafted the manuscript. CV-B, JW, and MW reviewed and edited the manuscript.

## Conflict of Interest

The authors declare that the research was conducted in the absence of any commercial or financial relationships that could be construed as a potential conflict of interest.

## Publisher’s Note

All claims expressed in this article are solely those of the authors and do not necessarily represent those of their affiliated organizations, or those of the publisher, the editors and the reviewers. Any product that may be evaluated in this article, or claim that may be made by its manufacturer, is not guaranteed or endorsed by the publisher.

## Footnotes

1 The authors will make all questionnaires that have been used in this study available upon request.

3
https://aspredicted.org/e6tr3.pdf

4
https://rdocumentation.org/packages/irr/versions/0.84.1

5 It must be noted that the positive association between sensibility scores and emotional granularity was exclusively observed for negative words. Although we did not predict a valence-specific effect, this finding converges with previous studies showing stronger associations between the granularity for negative words and external indicators (Barrett et al., 2001; Demiralp et al., 2012; Kashdan and Farmer, 2014; Kalokerinos et al., 2019). One potential reason for the divergence between the granularity for positive and negative words may be related to the fact that, at least in the current sample, granularity for positive words did not reflect the differentiation between emotional experiences to the same extent as granularity for negative words. In this line, we found that the granularity scores for negative words were significantly higher than for their positive counterparts (*t*[116] = 13.39, *p* < 0.001, *d* = 1.23), indicating that, when describing an event, the differentiation between negative words was higher than between positive ones. Additionally, and unlike previous studies (Barrett, 1998), in the current sample, we observed no significant association between both measures of granularity, suggesting that they might not be moderated by the same underlying process. Future studies examining the differences and similarities between granularity indices for positive and negative words could help understand the dissociation between them.
